# Dichloroacetate induces apoptosis and cell-cycle arrest in colorectal cancer cells

**DOI:** 10.1038/sj.bjc.6605701

**Published:** 2010-05-18

**Authors:** B M Madhok, S Yeluri, S L Perry, T A Hughes, D G Jayne

**Affiliations:** 1 Section of Translational Anaesthesia & Surgery, University of Leeds, Level 7 Clinical Sciences Building, St. James's University Hospital, Leeds, UK; 2 Leeds Institute of Molecular Medicine, University of Leeds, St. James's University Hospital, Leeds, UK

**Keywords:** dichloroacetate, colorectal cancer, pyruvate dehydrogenase, pyruvate dehydrogenase kinase

## Abstract

**Background:**

Cancer cells are highly dependent on glycolysis. Our aim was to determine if switching metabolism from glycolysis towards mitochondrial respiration would reduce growth preferentially in colorectal cancer cells over normal cells, and to examine the underlying mechanisms.

**Methods:**

Representative colorectal cancer and non-cancerous cell lines were treated with dichloroacetate (DCA), an inhibitor of pyruvate dehydrogenase kinase.

**Results:**

Dichloroacetate (20 mM) did not reduce growth of non-cancerous cells but caused significant decrease in cancer cell proliferation (*P*=0.009), which was associated with apoptosis and G_2_ phase cell-cycle arrest. The largest apoptotic effect was evident in metastatic LoVo cells, in which DCA induced up to a ten-fold increase in apoptotic cell counts after 48 h. The most striking G_2_ arrest was evident in well-differentiated HT29 cells, in which DCA caused an eight-fold increase in cells in G_2_ phase after 48 h. Dichloroacetate reduced lactate levels in growth media and induced dephosphorylation of E1*α* subunit of pyruvate dehydrogenase complex in all cell lines, but the intrinsic mitochondrial membrane potential was reduced in only cancer cells (*P*=0.04).

**Conclusions:**

Pyruvate dehydrogenase kinase inhibition attenuates glycolysis and facilitates mitochondrial oxidative phosphorylation, leading to reduced growth of colorectal cancer cells but not of non-cancerous cells.

Colorectal cancer is the third most common cancer in the world and the fourth leading cause of cancer-related death (Shike *et al*, 1990). In 2007 colorectal cancer accounted for 17.1 deaths per 100 000 persons in the United Kingdom (UK Bowel Cancer Statistics, 2009). Despite recent advances, the prognosis of patients with advanced and metastatic colorectal cancer remains poor. Targeting tumour metabolism for cancer therapy is a rapidly developing area (Pan and Mak, 2007). Early observations concerning the metabolic differences between cancer and normal cells were made by Otto Warburg, who showed that cancer cells are inherently dependent on glycolysis for production of chemical energy (Warburg, 1956). There is now mounting evidence that this increased glycolysis results from the influence of multiple molecular pathways, including adaptive responses to the hypoxic tumour microenvironment, oncogenic signalling, and mitochondrial dysfunction (Gatenby and Gillies, 2004; Gillies and Gatenby, 2007; Wu *et al*, 2007). The glycolytic phenotype offers growth advantages to cancer cells by resisting apoptosis, and facilitating tumour spread and metastasis (Yeluri *et al*, 2009).

A key regulator of cellular metabolism is pyruvate dehydrogenase (PDH). Pyruvate dehydrogenase converts pyruvate, produced from glycolysis, to acetyl-CoA, which is oxidised in the tricarboxylic acid cycle within mitochondria. Pyruvate dehydrogenase activity is tightly regulated by inhibitory phosphorylation by pyruvate dehydrogenase kinase (PDK). Phosphorylation occurs on the E1*α* sub-unit of PDH (PDHE1*α*) at three sites: Ser^232^, Ser^293^, and Ser^300^ (Rardin *et al*, 2009). Dichloroacetate (DCA) is an inhibitor of all the four isoenzymes of PDK(1–4) (Stacpoole, 1989), and has recently been shown to reduce growth of lung, endometrial, and breast cancer cell lines (Bonnet *et al*, 2007; Wong *et al*, 2008; Sun *et al*, 2009). It has been reported to reduce growth of these cancer cells mainly by reducing inhibitory phosphorylation of PDH, thereby promoting mitochondrial oxidative phosphorylation and inducing apoptosis through mitochondrial, NFAT-Kv 1.5, and p53 upregulated modulator of apoptosis (PUMA)-mediated pathways.

Colorectal cancer cells have been found to undergo increased glycolysis (Bi *et al*, 2006), and the tumour microenvironment has been found to be hypoxic and acidotic, mainly due to poorly developed blood supply (Dewhirst *et al*, 1989; Milosevic *et al*, 2004). We have previously shown that this is especially true for the more aggressive phenotype (Thorn *et al*, 2009), and expression of the important markers of hypoxia is increased in colorectal cancer especially at the invasive margin (Rajaganeshan *et al*, 2008, 2009). The purpose of this study was to investigate the effects of DCA on the growth of colorectal cancer cells in an attempt to examine PDK inhibition as a novel therapeutic strategy against colorectal cancer.

## Materials and methods

### Cell cultures

All cell lines were purchased from American Type Culture Collection (Manassas, VA, USA) or European Collection of Cell Cultures (Salisbury, Wiltshire, UK): HB2 (breast epithelial cells of non-cancer origin), 293 (epithelial cells from human embryo kidney), HT29 (well-differentiated primary colorectal adenocarcinoma), SW480 (poorly differentiated primary colorectal adenocarcinoma), and LoVo (metastatic left supraclavicular lymph node from colorectal adenocarcinoma). 293 and HB2 cells were maintained in DMEM medium, HT29 and SW480 in RPMI 1640 medium, and LoVo in F12 medium (all from Invitrogen, Carlsbad, CA, USA), supplemented with 10% fetal calf serum, in a 37°C, 5% CO_2_ humidified incubator. For experiments under hypoxic conditions, we incubated cells in a humidified, hypoxic incubator (1% O_2_, 5% CO_2_, 94% N_2_, 37°C). Sodium dichloroacetate (Specials Lab, Prudhoe, UK) was donated by the Pharmacy Department at St. James's University Hospital, Leeds, UK.

### MTT assays

Cells (1 × 10^4^) per well were seeded in 96-well tissue culture plates. After overnight incubation, we replaced media with fresh media containing increasing doses of DCA (0, 10, 15, 20, 30, 50, and 100 mM). After 24 and 48 h of incubation, we performed MTT assay by replacing the media with 50 *μ*l of 1 mg ml^−1^ MTT solution and the plates were incubated in the dark for 3 h. MTT solution was then removed and the dark blue formazan precipitates were dissolved in 100 *μ*l of propan-1-ol. Optical density was measured using microplate reader (Opsys MR; Dynex Technologies Ltd, Worthing, West Sussex, UK) at 570 nm.

### Annexin V and 7-AAD assays

Cells were seeded in 25 cm^2^ tissue culture flasks and incubated overnight in standard conditions. Media was replaced with fresh media containing a range of doses of DCA (0, 10, 20, and 50 mM). Flow cytometric analysis was performed after 24 and 48 h of incubation. Cells were washed twice with cold PBS and resuspended in 1 × binding buffer (BD Bioscience, Franklin Lakes, NJ, USA) at 5 × 10^6^ cells per ml. 100 *μ*l of solution (5 × 10^5^ cells) was transferred to 5 ml culture tubes. These cells were stained with 5 *μ*l annexin V-FITC and 10 *μ*l 7-AAD (BD Bioscience), gently vortexed, and incubated at ambient temperature for 15 min in dark. Following this 400 *μ*l 1 × binding buffer was added to each tube and analysed within an hour on LSR II flow cytometer (BD Bioscience).

### Propidium iodide assays

Cells were propagated as mentioned for the apoptosis assay. Dichloroacetate (50 mM) was used and compared to vehicle control. After harvesting, we resuspended cells in 350 *μ*l of PBS at a concentration of 0.5–1.0 × 10^6^ cells per ml. 100 *μ*l of 0.25 mg ml^−1^ propidium iodide (PI)/5% Triton (Sigma, St Louis, MO, USA) was added to the cell suspension. 50 *μ*l of 1 mg ml^−1^ ribonuclease A (Sigma) was then added. Sample tubes were thoroughly vortexed and incubated for 10 min in the dark at room temperature. Flow cytometry was performed on LSR II flow cytometer (BD Bioscience) and data were analysed using FlowJo software (FlowJo, Ashland, OR, USA).

### Lactate measurements

Lactate measurements in growth media were performed by the chemical pathology department at the General Infirmary, Leeds Teaching Hospitals NHS Trust. Cells were incubated in 25 cm^2^ flasks overnight in normoxia. Media was replaced next day with a range of doses of DCA (0, 10, 20, and 50 mM). After 48 h of incubation, we collected 2 ml of media in fluoride tubes and transferred immediately to the chemical pathology laboratory. The tubes were maintained on ice during the transfer. Lactate levels were measured using an automated analyser (Advia 1200 Chemistry system; Siemens Healthcare Diagnostics, Camberley, Surrey, UK).

### TMRM assays

Cells were treated with DCA as described for the apoptosis assay. After 24 and 48 h of incubation, we washed cells in PBS, and suspended 1 × 10^6^ cells per ml in Hank's buffered salt solution with 50 nM tetramethylrhodamine methyl ester (TMRM) (Invitrogen). 100 *μ*l of the cell suspension (1 × 10^5^ cells per well) was transferred to opaque 96-well plates, incubated for 30 min, and fluorescence was measured at 530/620 nm at 37°C using a plate reader (Mithras LB 40; Berthold Technologies, Bad, Wildbad, Germany).

### Western blotting

Cells were treated with DCA as described above. After 8 h of treatment, we extracted proteins from cells in Laemmli buffer (2% SDS, 10% glycerol, 0.7% 2-mercaptoethanol, 0.05% bromophenol blue, and 0.5 M Tris-HCl). Lysates were resolved by electrophoresis on NuPAGE Novex 12% Bis-Tris gels (Invitrogen) in MOPS-SDS running buffer (Invitrogen). Proteins were transferred to a polyvinylidene fluoride membrane (GE Healthcare, Chalford St Giles, Bucks, UK). The membrane was blocked for 1 h at ambient temperature in 5% skimmed milk in TBS-T (Tris-buffered saline with 0.1% Tween). The membrane was then probed with primary antibodies in 1% skimmed milk in TBS-T for 90 min, washed in TBS-T, and then probed with the appropriate horseradish peroxidase (HRP)-conjugated secondary antibody for 60 min. Primary antibodies rabbit polyclonal phosphodetect anti-PDH-E1*α* (pSer^293^), 1 : 500 (AP1062; EMD Chemicals, Darmstadt, Germany), and mouse monoclonal anti-PDHE1*α*, 1 : 500 (459400; Invitrogen). Secondary antibodies anti-rabbit or anti-mouse HRP conjugates, 1 : 1000 (Dako, Glostrup, Denmark). Proteins were visualised with Supersignal West Pico or Femto chemiluminescent substrate (Pierce Biotechnology, Rockford, IL, USA) and the Chemidoc XRS system (Bio-Rad, Hercules, CA, USA). *β*-Actin was used as a loading control.

### Statistical analyses

Flow cytometry data were acquired using specific software, BD FACSDiva 6.0 and FlowJo software. Statistical analyses were performed using SPSS for Windows (SPSS version 15.0, Chicago, IL, USA). Differences between DCA-treated and vehicle control groups were assessed using the Mann–Whitney *U*-test and the 95% confidence intervals of the difference in means between the two groups. A *P*-value of less than 0.05 was considered to be statistically significant. Data are represented as mean from at least three independent experiments and error bars represent standard deviation of mean.

## Results

### DCA reduces cancer cell proliferation and the effect is similar in normoxia and hypoxia

First, we wished to determine if treatment with DCA inhibited cellular proliferation and whether there would be a differential response in cancer and non-cancerous cells in normoxic and hypoxic conditions. With respect to hypoxia, our hypothesis was that influence of DCA would be particularly potent with oxygen levels that are insufficient to support additional oxidative phosphorylation. All cell lines (HB2, 293, HT29, SW480, and LoVo) were treated with a range of doses of DCA for 24–48 h in normoxic and hypoxic conditions. Relative cell numbers were assessed using MTT assays.

**Figure 1:**
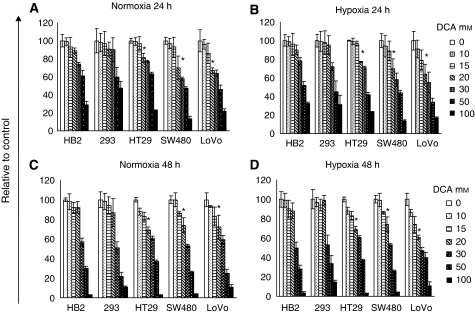
Dichloroacetate (20 mM) did not significantly reduce growth of cultures of the non-cancerous 293 and HB2 cells but caused a significant reduction in growth of cultures of all the colorectal cancer cells (^*^P⩽0.009). Cells were treated with various doses of DCA or vehicle control in normoxia (**A** and **C**) or hypoxia (**B** and **D**), and the relative number of viable cells was assessed at 24 h (**A** and **B**) and 48 h (**C** and **D**) using MTT assay. Data are expressed as percentage of control (0 mM dose) (^*^ – significant difference relative to control – white bar (0 mM)).

Treatment with increasing doses of DCA reduced cellular proliferation in a dose-dependent manner (Figure 1A-D). Contrary to our expectation, the profiles of reduced cell growth were similar in hypoxia and normoxia. At 24 and 48 h, up to 20 mM DCA did not affect the growth of cultures of the non-cancerous cells, HB2 and 293. However, 20 mM DCA significantly reduced growth of cultures of all three colorectal cancer cell lines (*P*⩽0.009). The effect of DCA was greater on the poorly differentiated SW480 cells and the metastatic LoVo cells than the well-differentiated HT29 cells. The growth of cultures of LoVo cells treated with 20 mM DCA was reduced by up to 40% compared to cells treated with vehicle control. Because there was relatively little difference in the reduction of growth of cultures treated with DCA in hypoxic and normoxic conditions, further experiments were performed only in normoxia.

### DCA promotes apoptosis in cancer cells sparing non-cancerous cells

**Figure 2:**
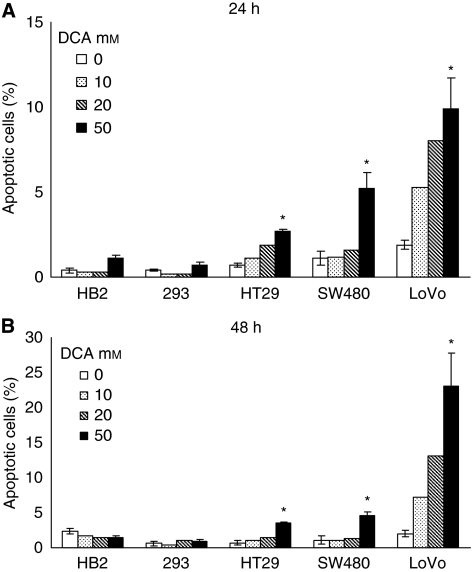
Dichloroacetate induced a dose-dependent increase in percentage of the apoptotic population in the cancer cells with minimal apoptosis in the non-cancerous cells. Cells were treated with doses of DCA for 24 h (**A**) and 48 h (**B**), stained with annexin V-FITC and 7-AAD, and analysed with flow cytometry. Data points represent the mean (±s.d.) of three independent experiments for 0 and 50 mM DCA (^*^ – significant difference relative to control).

Next, we wished to investigate whether the reduced growth of cultures on treatment with DCA was associated with induction of apoptosis. Cells were treated with range of doses of DCA (0, 10, 20, and 50 mM) for 24 and 48 h, and the proportion of cells undergoing apoptosis was assessed by detecting membrane phosphatidylserine with annexin V-FITC. Cells were stained with annexin V-FITC and vital dye 7-AAD, and analysed using flow cytometry. There was a dose-dependent induction of apoptosis in the cancer cell lines after 24 and 48 h of treatment, with little, if any, apoptosis induced in the non-cancerous cells (Figure 2A and B). The greatest effect was observed in the metastatic LoVo cells; 50 mM DCA caused a ten-fold increase in the proportion of apoptotic cells after 48 h, whereas there was a seven- and five-fold increase in HT29 and SW480 cells, respectively. Increase in the mean percentage of total apoptotic cells with 50 mM DCA was: 2.8 (95% CI: 2–3) in HT29 cells, 3.5 (95% CI: 2–5) in SW480 cells, and 21 (95% CI: 8–34) in LoVo cells. There was minimal apoptosis induced in the 293 cells even with 50 mM DCA, 0.2 (95% CI: −0.2 to 0.6). In HB2 cells, there was a nonsignificant decrease in the percentage of apoptotic cells on treatment with 50 mM DCA, −0.9 (95% CI: −2.2 to 0.4).

### DCA induces G_2_ phase arrest in colorectal cancer cells but has no effect on cell-cycle profile of non-cancerous 293 cells

**Figure 3:**
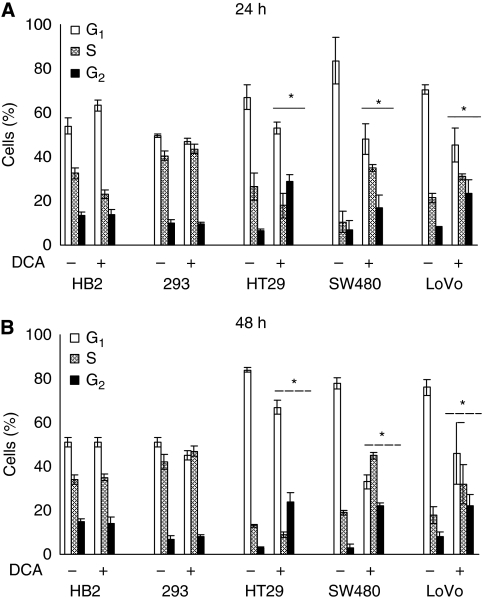
Dichloroacetate induced G_2_ phase arrest in colorectal cancer cells with no effect on cell-cycle profiles of non-cancerous cells; 293 and HB2. Cells were treated with 50 mM DCA or vehicle control for 24 h (**A**) and 48 h (**B**), stained with PI, and analysed with flow cytometry. For analyses of statistical significances, we compared mean proportion of cells in each phase of cell cycle (G_1_, S, and G_2_) in DCA-treated cells to mean proportion of cells in the respective phases in untreated cells (^*^ – significant difference relative to control).

We also wished to examine whether the reduction in growth of cultures on treatment with DCA was associated with induction of growth arrest. Cells were treated with 50 mM DCA for 24 or 48 h, and cell-cycle profiles were analysed using flow cytometric assessment of DNA content after PI staining. Dichloroacetate treatment caused changes in the cell-cycle profiles of all the cancer cells but did not affect the non-cancerous cells. The changes in cell-cycle profile were detectable after 24 h of treatment, and were persistent at 48 h (Figure 3A and B).

After 48 h of treatment with 50 mM DCA, there was an eight-fold increase in the cells in G_2_ phase in HT29 and SW480 cells, and three-fold increase in LoVo cells. Increase in the mean percentage of all cancer cells in G_2_ phase was: 21 (95% CI: 13–30) for HT29, 19 (95% CI: 13–24) for SW480 cells, and 14 (95% CI: 10–21) for LoVo cells; whereas there was no difference in the 293 cells, 1 (95% CI: −4 to 7), and HB2 cells, −0.3 (95% CI: −9 to 9). There was a corresponding decrease in cells in G_0_/G_1_ phase in all cancer cell lines. Intriguingly, in HT29 cells there was a small decrease, but in SW480 and LoVo cells there was a significant increase in the proportion of cells considered to be in the S phase (see Discussion section). The cell-cycle profile of 293 and HB2 cells changed minimally on treatment with DCA.

### DCA reduces extracellular lactate levels in growth media

**Figure 4:**
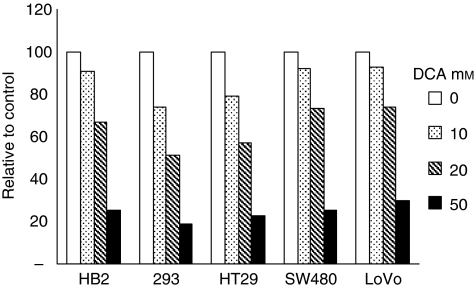
Dichloroacetate reduced lactate levels in growth media in a dose-dependent manner in both cancer and non-cancerous cells. Cells were treated with range of doses of DCA for 48 h, and extracellular lactate levels were measured in the growth media using an auto-analyser. Results are expressed as relative of control.

To establish whether the changes in growth and apoptosis induced by DCA correlated with reduced glycolysis, we measured lactate levels in growth media. Lactic acid is the end product of glycolysis. If DCA were inducing mitochondrial oxidative phosphorylation, pyruvate would be decarboxylated to acetyl-CoA and not reduced to lactate, hence lactate levels in the growth media would decrease. Lactate levels in growth media of all cell lines were measured after 48 h of treatment with a range of doses of DCA (Figure 4). Lactate levels were determined with an auto-analyser that is used routinely for biochemical measurement of lactate levels; the assays are based on a colorimetric reaction catalysed by lactate oxidase. Treatment with DCA reduced extracellular lactate levels in growth media in a dose-dependent manner in all the cancer and non-cancerous cell lines.

### DCA depolarises the intrinsic mitochondrial membrane in colorectal cancer cells but not in non-cancerous cells

To verify if the induction of apoptosis in cancer cells on treatment with DCA was associated with promotion of mitochondrial oxidative phosphorylation, we measured the intrinsic mitochondrial membrane potential (ΔΨm). Escalation of mitochondrial respiration would reactivate the electron transport chain and reduce the hyperpolarised ΔΨm in cancer cells. Cells were treated with doses of DCA for 24 and 48 h and stained with the dye TMRM, which allows fluorescent measurement of ΔΨm.

**Figure 5:**
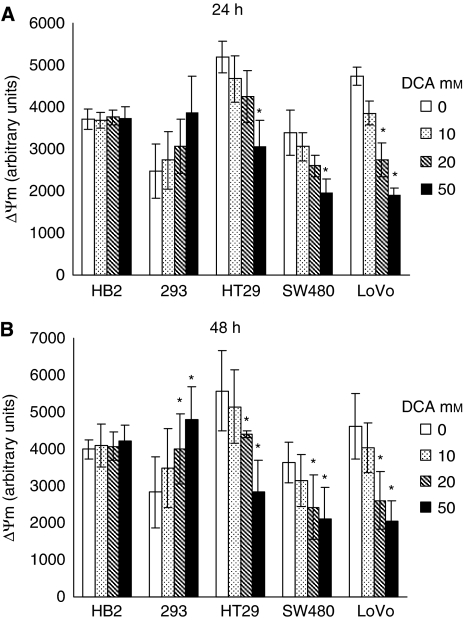
Dichloroacetate treatment reduced the intrinsic mitochondrial membrane potential (ΔΨm) in all cancer cells, increased ΔΨm in non-cancerous 293 cells, and had no effect on ΔΨm in non-cancerous HB2 cells. Cells were treated with doses of DCA for 24 h (**A**) and 48 h (**B**), stained with TMRM, and fluorescence was measured at 530/620 nm at 37°C (^*^ – significant difference relative to control).

As with previous experiments, the effect of DCA was apparent after 24 h of treatment and persisted at 48 h (Figure 5A and B). Dichloroacetate treatment reduced the hyperpolarised ΔΨm in all the cancer cells in a dose-dependent manner. Dichloroacetate did not have any effect on ΔΨm of the non-cancerous HB2 cells, whereas, surprisingly the ΔΨm of the non-cancerous 293 cells increased in a dose-dependent manner. At 24 h of treatment, 50 mM DCA significantly reduced ΔΨm in all cancer cells; however, in LoVo cells there was a significant reduction even with 20 mM DCA (Figure 5A, *P*=0.02). In the non-cancerous 293 cells, there was a trend towards increase in ΔΨm on DCA treatment, although this was not statistically significant (*P*=0.08). At 48 h of treatment, there was significant reduction of ΔΨm in all cancer cells and increase in the 293 cells, with 20–50 mM DCA (Figure 5B, *P*⩽0.04).

### DCA treatment leads to dephosphorylation of the PDHE1*α* sub-unit

**Figure 6:**
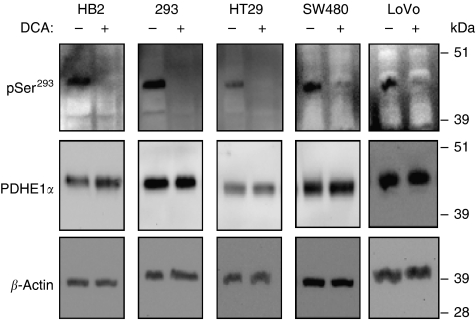
Dichloroacetate treatment reduced phosphorylation of PDHE1*α* at pSer^293^ site with no effect on the levels of total PDHE1*α* in all the cell lines investigated. Whole-cell lysates were prepared after treating cells with 20 mM DCA for 8 h and from untreated cells, and western blot analyses were performed.

DCA is thought to inhibit all four isoenzymes of PDK, and hence reduce phosphorylation of the PDHE1*α* sub-unit, leading to, in turn, activation of the PDH complex. To verify if the dephosphorylation of PDHE1*α* was occurring with DCA treatment in the cell lines used, we used western blot analyses on lysates of DCA-treated and untreated cells. In all cell lines, treatment with 20 mM DCA for 8 h caused a dramatic reduction in signal for phosphorylation at the pSer^293^ site, but no change was detected in the levels of total PDHE1*α* (Figure 6). Phospho-specific antibodies for the other two phosphorylation sites, Ser^232^ and Ser^300^, are not yet commercially available.

## Discussion

### Differential effects of DCA on growth of cancer and non-cancerous cells

We have shown that DCA induces a dose-dependent reduction in growth of *in vitro* cultures of colorectal cancer cells and non-cancerous cells. However, the cancer cells were more sensitive to DCA, with a dose of 20 mM causing a significant inhibition of cancer cell growth, but having little effect on the non-cancerous cells. We have shown that the components of this differential effect are the following: a potent induction of apoptosis and cell-cycle arrest in cancer cells, but not in the non-cancerous cells.

These conclusions support a simple model of differential sensitivity to DCA. However, some data require further discussion. First, 50 mM DCA reduced growth of cultures of the non-cancerous 293 and HB2 cells, yet no increase in apoptotic cells or change in cell-cycle profile of these cells was observed. A possible explanation for these findings could be that this dose of DCA led to a slower transit of these non-cancerous cells through all stages of the cell cycle, without changing the relative proportions within each stage. Second, our results indicate that DCA induced G_2_ arrest in colorectal cancer cells. This is in contrast to previous studies, which have shown G_1_ arrest or no change on cell-cycle profile with DCA treatment (Cao *et al*, 2008; Wong *et al*, 2008). Wong *et al* (2008) showed increased expression of PUMA in all the endometrial cancer cell lines that had an apoptotic response to DCA, and concluded that this p53 activation led to G_1_ arrest. However, colorectal cancer cells in our study arrested in G_2_ phase on treatment with DCA, and we did not find any induction of p53 by DCA in our colorectal cancer cell lines (data not shown). Intriguingly, Cao *et al* (2008) found that the combination of DCA and radiotherapy arrested prostate cancer cells in G_2_ phase, although DCA on its own did not affect cell-cycle profile. Third, in SW480 and LoVo cells, DCA treatment resulted in an increase in the proportion of cells considered to be in the S phase. This suggests an increase in proliferation as well as induction of apoptosis. A similar finding was reported by Wong *et al* (2008) in one of several endometrial cancer cells tested. An alternative explanation is that a proportion of the cells observed to be in ‘S phase’ after DCA treatment of the cancer cell lines actually represent apoptotic cells in the ‘sub-G_2_’ region, as has been reported previously in lymphoma cells (Klucar and Al-Rubeai, 1997).

### Changes in cellular metabolism with DCA treatment

DCA appeared to suppress lactic acid production from pyruvate in both cancer and non-cancerous cells. In addition, treatment with DCA led to dephosphorylation of PDHE1*α*, and hence activation of PDH in all the cell lines investigated. Hence, the basis of DCA's differential effect on cancer and non-cancerous cells may reside in its influence on mitochondrial function. Treatment with DCA reduced the high ΔΨm of all cancer cells but not of the non-cancerous cells. This suggests that DCA, by inhibiting PDK and hence activating PDH, promotes mitochondrial respiration that leads to depolarisation of the intrinsic mitochondrial membrane, and induces apoptosis by the proximal mitochondrial pathway as described in the previous studies (Bonnet *et al*, 2007; Cao *et al*, 2008; Wong *et al*, 2008). Induction of apoptosis and changes in mitochondrial function were most pronounced in the highly invasive and metastatic LoVo cells than the less invasive HT29 and SW480 cells. This could have clinical implications for the treatment of metastatic colorectal cancer, as it is usually the highly invasive metastatic cancers that are most resistant to conventional chemotherapy, and which may be most sensitive to PDK inhibition. In support of this, a recent study reported that the colorectal tumours resistant to 5-fluorouracil are more likely to have upregulated glycolysis, and hence more amenable to therapy targeting cancer metabolism (Shin *et al*, 2009). In this regard, our results contrast the findings of Wong *et al* (2008), who found highly invasive endometrial cancer cells to be most resistant to DCA treatment.

### PDK inhibition as cancer therapy against colorectal cancer

We found doses of 20–50 mM DCA gave differential responses between cancer and non-cancerous cells. Thus, potential therapeutic DCA doses would be between 20 and 50 mM. In addition, a recent study reported that the IC_50_ of DCA for breast cancer cells to be between 20 and 30 mM (Ko and Allalunis-Turner, 2009). This is in contrast to previous studies that have reported DCA to reduce proliferation and induce apoptosis in cancer cells with doses as low as 0.5–10 mM (Bonnet *et al*, 2007; Wong *et al*, 2008; Sun *et al*, 2009). Dichloroacetate has been found to be relatively safe in humans when used for treatment of lactic acidosis (Stacpoole *et al*, 2003). The main side effects with up to 100 mg kg^−1^ DCA are on the nervous system and the liver, causing mild sedation or drowsiness, reversible peripheral neuropathy, and mild asymptomatic elevation of serum transaminases reflecting hepatocellular damage (Stacpoole *et al*, 1998). In addition, recent studies reported that DCA effectively reduced tumour growth in clinically achievable doses both *in vitro* and *in vivo* (Bonnet *et al*, 2007; Sun *et al*, 2009). It was suggested that DCA could rapidly translate to early-phase cancer clinical trials (Michelakis *et al*, 2008). However, the dose of DCA required to inhibit growth of colorectal cancer cells in our study is unlikely to be achieved clinically without causing significant side effects. The dose of DCA required to achieve the equivalent plasma concentrations *in vivo* would be about five to ten times than that used in clinical trials against lactic acidosis. It appears that the colorectal cancer cells used in our study are more resistant to DCA than lung, endometrial, and breast cancer cells. Intriguingly, Sun *et al* (2009) in their study on breast cancer cells found that DCA inhibited proliferation of cancer cells, but did not induce apoptosis or cell death. These results were markedly different to the effects of DCA observed on lung (Bonnet *et al*, 2007), endometrial (Wong *et al*, 2008), and colorectal cancer cells in our study. Thus, although DCA inhibits growth of a variety of cancer cells, the effect and the underlying mechanisms seem to be cell-type dependent. A likely explanation for these differential effects could be the difference in expression of the PDK isoenzymes in the cancer cells examined. Dichloroacetate is a non-specific inhibitor of PDK (Whitehouse and Randle, 1973), and has a different *K*_i_ for each of the four PDK isoenzymes (Bowker-Kinley *et al*, 1998). In addition, the four PDK isoenzymes are known to be differentially expressed in various tissues. Thus, there is a need to develop inhibitors to the individual PDK isoenzymes that should allow cancer cell-type-specific metabolic manipulation.
